# Using genAI in education: the case for critical thinking

**DOI:** 10.3389/frai.2024.1452131

**Published:** 2024-11-01

**Authors:** Chien Ching Lee, Malcolm Yoke Hean Low

**Affiliations:** ^1^Centre for Professional Communication, Singapore Institute of Technology, Singapore, Singapore; ^2^Information and Communication Technology Program, Singapore Institute of Technology, Singapore, Singapore

**Keywords:** genAI, education, critical thinking, empowerment, ownership

## 1 Introduction

With AI technologies driven by big data, many claim that we are now experiencing a global Fourth Industrial Revolution (MacGregor, 2024). Countries like Singapore (Chia, 2023) and America (MacGregor, 2024) are investing and rushing to develop AI talent and solutions. If one of the main roles of universities is to equip students for jobs of the future, there is no turning back from training students in the proficient and ethical use of generative AI (genAI). As mentioned by Heaven (2023), genAI tools are changing education, not destroying it.

United Nations Educational Scientific Cultural Organization (2023) has formulated guidance for policymakers on AI and education which has helped institutions of higher learning navigate the use of genAI. Research has found that university staff are aware that the use of genAI could result in better productivity and hence a majority are optimistic about its use (McCormack, 2023). Students are also aware of the benefits and challenges faced in the use of genAI. They are drawn to the personalized feedback that genAI could offer 24/7, features which are useful with increasing class sizes (Dixon, 2023). Kakuchi's (2023) study further found that 78% and 70% of students mentioned that genAI helped improve their writing and thinking, respectively. However, they worry about unintentionally running afoul of university guidelines on its use as it becomes integrated in most software tools (Hodges and Ocak, 2023) and how an overreliance on genAI could influence the value of their university education (Chan and Hu, 2023).

This opinion piece argues that as AI eventually becomes ubiquitous in education, educators should aim to use the technology to challenge students to think critically and enhance their human interactions. This argument is supported by the findings from the World Economic Forum's (2023) Future of Work report where creative thinking, analytical thinking and self-efficacy skills were rated as the top three skills.

Two use cases from the Singapore Institute of Technology (SIT), an applied learning university, are presented. The first case relates to communication (soft) skills classes taught by the first author while the second case relates to information technology (hard skills) taught by the second author. The two cases reflect SIT's position that genAI tools could enhance teaching and learning, and perhaps industry practices. Thus, students are encouraged to use genAI as tools rather than as primary sources of information (Rakshika and Lee, 2024). This aligns with the Singapore government's initiative for students to be taught the ethical and responsible use of AI at different levels (Ang, 2024) and its Draft GenAI Governance Framework which aims to promote a systemic and balanced approach to facilitate innovation and address accountability issues in the use of genAI tools (Norton Rose Fulbright, 2024).

## 2 Small classes teaching soft skills

Soft skills like communication skills are often taught seminar style in the classroom. In SIT, undergraduate students are taught to think critically and connectedly. Critical thinking is taught using the Paul-Elder framework (Paul and Elder, 2019) in a core common module “Critical Thinking and Communicating” in year 1 and is reinforced in the Communicating Across the Curriculum (CAC) embedded workshops throughout their studies. The Paul-Elder framework aims to inculcate intellectual elements that help students question issues from multiple perspectives, while the intellectual standards focus on checking the quality of students' reasoning. Recently, students have also been taught systems thinking (Thinking Tools Studio, 2024) in some workshops to enhance their understanding on how issues are interrelated.

Among the CAC workshops taught are Writing the Literature Review, Logbook Writing and Revising Penetration Testing Reports. The workshops catered to 119, 30, and 119 students respectively from the Information Technology program in the second trimester of 2023. The materials were developed based on the assignment brief provided by the respective module leads. The workshops were 3, 6, and 3 hours long respectively and taught via zoom.

The innovations in these workshops are the intentional teaching on how to use and evaluate outputs from ChatGPT 3.5 (a free genAI tool) using the Paul-Elder framework and systems thinking habits. The instructor demonstrated how to use ChatGPT on a parallel task in the workshop activities by teaching them how to use ChatGPT for various tasks related to the workshops, and providing them with feedback on the revisions needed in ChatGPT's outputs using the Paul-Elder framework. Then, the students performed a similar task using ChatGPT in groups of four to five in zoom breakout rooms. The students uploaded their drafts to the discussion forum in the LMS and the instructor led feedback-cum-questioning sessions with their peers on a few of the students' drafts. For the logbook writing workshop, the students were also taught the 14 systems thinking habits aided by prompts from the flip cards (Thinking Tools Studio, 2024). The aim was to help students understand the implications of their decisions in the larger and dynamic contexts of a company and/or society.

The feedback from the students was positive. The online surveys conducted at the end of the workshops showed that students were more aware about how to be strategic and critical when using ChatGPT in their learning. They liked the efficiency afforded by ChatGPT even though they needed to edit ChatGPT's outputs. They also put in more effort to include key points in their writing which pre-empted questions from the instructor and their peers in class. The students also mentioned that they have gained more confidence in navigating the use of genAI tools for future assignments. Interestingly, even though the systems thinking habits were taught briefly in the logbook writing workshop, it resonated with the students, with 10 out of the 14 habits cited in the survey. These outcomes reflected a positive and interactive learning environment, aided by discussions and questioning on ChatGPT's outputs.

## 3 Large classes teaching hard skills

Teaching data structures and algorithms (DSA) presents significant challenges in computer science education due to the abstract and complex nature of these foundational topics. Students often struggle to grasp the theoretical underpinnings and practical applications of DSA, which are crucial for effective problem-solving in various technological fields. Instructors face the difficult task of conveying these intricate concepts in a manner that is both engaging and comprehensible, particularly given the diverse learning paces and styles of students.

In response to these challenges, we integrated AI chatbots into the Data Structures and Algorithms (DSA) course, which is a core component of several undergraduate degree programs. The course was delivered to a cohort of 178 students in the second trimester of 2023. It covered fundamental topics such as algorithm analysis, different data structures like stacks, queues, and trees, and algorithms for sorting, searching, and optimizing paths in graphs.

A significant innovation in the course was the inclusion of a team project that required students to use AI chatbots as a learning tool. This project was introduced in the latter half of the course and aimed to enhance students' understanding of DSA through interaction with chatbots. Students were grouped into teams of four to five, and each team selected a specific data structure or algorithm to focus on. They interacted with AI chatbots to ask questions and assess the responses based on accuracy, completeness, clarity, and relevance. The assignment also required students to propose a real-world application for their chosen topic and document their findings and interactions in a detailed report.

The feedback from the students on the use of AI chatbots in the team project was largely positive. Surveys conducted at the end of the trimester showed high ratings for the accuracy, clarity, and relevance of the chatbot responses. Students appreciated the interactive nature of the chatbots, noting that this approach made the learning process more engaging and contributed to a better understanding of complex DSA topics. The introduction of AI chatbots not only provided a more interactive and personalized learning experience but also sparked a deeper reflection among students on the human aspects of learning, such as creativity, highlighting a clear distinction in educational engagement compared to traditional methods without such technology. These outcomes suggest that AI chatbots, with further refinement, could become invaluable educational tools in computer science, helping to mitigate some of the traditional challenges associated with teaching DSA effectively.

## 4 Discussion

The current generation Z are digital natives. Thus, they take to genAI like fish to the water. Coffey (2023) further found that genAI is used daily by 50% of college students in helping them with their school assignments, with the number of users set to increase year on year. In addition, 30% of the students believe that they need to be well-versed in its use to gain employment. We have shared two use cases which demonstrated how genAI was used to promote students' curiosity and accountability and which empowered their humanity. We would like to suggest that instructors need to model the way in which students could use genAI meaningfully and ethically. Another suggestion could be to utilize formative assessments with clear guidelines for the use of genAI which focuses on the learning process rather than the product *per se*. Chan's (2023) 3R framework encourages students to Report their use of genAI tools, Revise its output and Reflect on the process. This framework recognizes AI-assisted writing as basic writing which leads to specific writing purposes. Having students declare the originality of their submissions could be another ethical safeguard as it has mostly been effective in deterring academic dishonesty (Borup, 2023). Instructors have often wished for teaching assistants to help them manage increasing class sizes. With proper guidance, genAI, including recent advanced reasoning models such as OpenAI o1, could serve students in that role, offering students an interactive and personalized learning environment for their learning and possibly equip them for the future of work which utilizes genAI.

## References

[B1] Ang S. (2024). Students Are Taught to Use AI Ethically and Responsibly at Different Levels: Chan Chun Sing. Available at: https://www.straitstimes.com/singapore/politics/students-are-taught-to-use-ai-ethically-and-responsibly-at-different-levels-chan-chun-sing (accessed September 20, 2024).

[B2] Borup J. (2023). This Was Written By a Human: a Real Educator's Thoughts on Teaching in the Age of Chatgpt. EDUCAUSE Review. Available at: https://er.educause.edu/articles/2023/3/this-was-written-by-a-human-a-real-educators-thoughts-on-teaching-in-the-age-of-chatgpt (accessed September 3, 2024).

[B3] Chan C. B. (2023). “Grading generative AI-based assignments using a 3R framework,” in Proceedings for IEEE International Conference on Teaching, Assessment and Learning for Engineering, 28 Nov-−1 Dec. Auckland.

[B4] Chan C. K. Y. Hu W. J. (2023). Students' voices on generative AI: perceptions, benefits and challenges in higher education. Int. J. Educ. Technol. High. Educ. 20:8. 10.1186/s41239-023-00411-8

[B5] Chia O. (2023). National AI Strategy 2.0 Follows Years of Planning, Growth in AI Sector “Not By Chance.” The Straits Times. Available at: https://www.straitstimes.com/singapore/national-ai-strategy-20-follows-years-of-planning-growth-in-ai-sector-not-by-chance-dpm-wong (accessed September 20, 2024).

[B6] Coffey L. (2023). Students outrunning faculty in AI Use. Inside Higher Education. Available at: https://www.insidehighered.com/news/tech-innovation/artificial-intelligence/2023/10/31/most-students-outrunning-faculty-ai-use (accessed January 29, 2024).

[B7] Dixon J. (2023). Keeping Human Values at the Heart of AI in Higher Education. University World News. Available at: https://www.universityworldnews.com/post.php?story=20230814140706902 (accessed January 29, 2024).

[B8] Heaven W. D. (2023). ChatGPT Is Going to Change Education, Not Destroy It. MIT Technology Review. Available at: https://www.technologyreview.com/2023/04/06/1071059/chatgpt-change-not-destroy-education-openai/ (accessed January 29, 2024).

[B9] Hodges C. Ocak C. (2023). Integrating Generative AI Into Higher Education: Considerations. EDUCASE Review. Available at: https://er.educause.edu/articles/2023/8/integrating-generative-ai-into-higher-education-considerations (accessed January 29, 2024).

[B10] Kakuchi S. (2023). Universities Take Lead in Forging AI Policies, Practices. University World News. Available at: https://www.universityworldnews.com/post.php?story=20230822082741187 (accessed September 3, 2024).

[B11] MacGregor K. (2024). Generative AI Action Hints at Core Future Roles in Universities. University World News. Available at: https://www.universityworldnews.com/post.php?story=2024012705101220 (accessed September 3, 2024).

[B12] McCormack M. (2023). EDUCAUSE QuickPoll Results: Adopting and Adapting to Generative AI in Higher Ed Tech. EDUCAUSE Review. Available at: https://er.educause.edu/articles/2023/4/educause-quickpoll-results-adopting-and-adapting-to-generative-ai-in-higher-ed-tech (accessed January 29, 2024).

[B13] Norton Rose Fulbright L. L. P. (2024). Singapore Proposes Governance Framework for Generative AI. Available at: https://www.dataprotectionreport.com/2024/02/singapore-proposes-governance-framework-for-generative-ai/ (accessed January 29, 2024).

[B14] Paul R. Elder L. (2019). The Miniature Guide to Critical Thinking Concepts and Tools, 8th Edn. Lanham, MD: Rowman & Littlefield Publishers; Foundation for Critical Thinking Press.

[B15] Rakshika V. Lee E. (2024). Students at Singapore Universities Allowed to Use AI Tools for Assignments But Must Stick to Rules. Available at: https://www.straitstimes.com/singapore/students-at-s-pore-universities-allowed-to-use-ai-tools-for-assignments-but-must-stick-to-rules (accessed January 29, 2024).

[B16] Thinking Tools Studio (2024). Habits of a Systems Thinker. Available at: https://thinkingtoolsstudio.waterscenterst.org/cards (accessed September 20, 2024).

[B17] United Nations Educational Scientific and Cultural Organization (2023). ChatGPT and Artificial Intelligence in Higher Education: Quick Start Guide. Available at: https://www.iesalc.unesco.org/wp-content/uploads/2023/04/ChatGPT-and-Artificial-Intelligence-in-higher-education-Quick-Start-guide_EN_FINAL.pdf (accessed January 29, 2024).

[B18] World Economic Forum (2023). Future of Jobs Report. Available at: https://www.weforum.org/reports/the-future-of-jobs-report-2023/ (accessed January 29, 2024).

